# Telomere Dynamics in Immune Senescence and Exhaustion Triggered by Chronic Viral Infection

**DOI:** 10.3390/v9100289

**Published:** 2017-10-05

**Authors:** Marcia Bellon, Christophe Nicot

**Affiliations:** Department of Pathology, Center for Viral Pathogenesis, University of Kansas Medical Center, Kansas City, KS 66160, USA; mbellon@kumc.edu

**Keywords:** HTLV, HIV, EBV, HBV, HCV, HDV, HHV-8, HPV, HSV, VZV, telomere, telomerase, exhaustion, senescence

## Abstract

The progressive loss of immunological memory during aging correlates with a reduced proliferative capacity and shortened telomeres of T cells. Growing evidence suggests that this phenotype is recapitulated during chronic viral infection. The antigenic volume imposed by persistent and latent viruses exposes the immune system to unique challenges that lead to host T-cell exhaustion, characterized by impaired T-cell functions. These dysfunctional memory T cells lack telomerase, the protein capable of extending and stabilizing chromosome ends, imposing constraints on telomere dynamics. A deleterious consequence of this excessive telomere shortening is the premature induction of replicative senescence of viral-specific CD8+ memory T cells. While senescent cells are unable to expand, they can survive for extended periods of time and are more resistant to apoptotic signals. This review takes a closer look at T-cell exhaustion in chronic viruses known to cause human disease: Epstein–Barr virus (EBV), Hepatitis B/C/D virus (HBV/HCV/HDV), human herpesvirus 8 (HHV-8), human immunodeficiency virus (HIV), human T-cell leukemia virus type I (HTLV-I), human papillomavirus (HPV), herpes simplex virus-1/2 (HSV-1/2), and Varicella–Zoster virus (VZV). Current literature linking T-cell exhaustion with critical telomere lengths and immune senescence are discussed. The concept that enduring antigen stimulation leads to T-cell exhaustion that favors telomere attrition and a cell fate marked by enhanced T-cell senescence appears to be a common endpoint to chronic viral infections.

## 1. Replicative Senescence in Chronic Viral Infection

**Figure 1:**
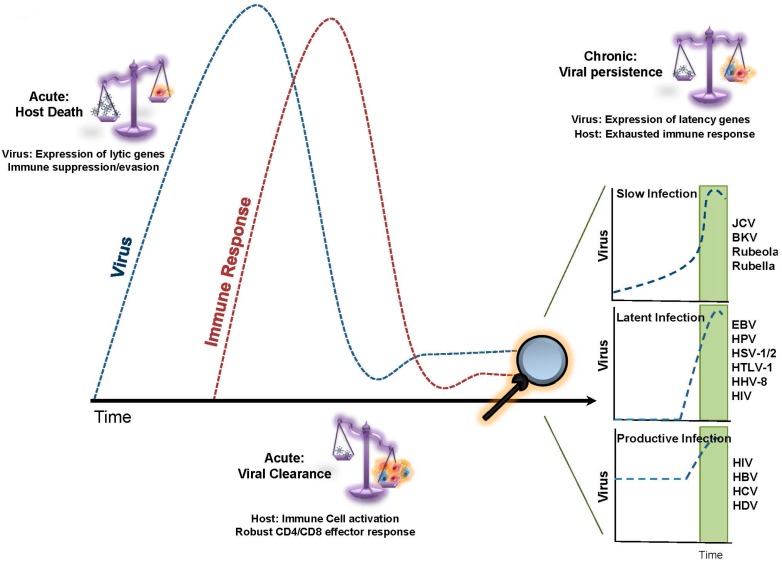
The relationship between host immune response and the invading virus during the course of acute or chronic viral infection. During acute viral infection, the balance swings in favor of viral production, leading to the expression of viral genes and rapid viral replication. The conclusion often involves either host death (enhanced viral replication; dotted blue line) or viral clearance (enhanced immune response; dotted red line). The latter involves a robust immune effector response from CD4+ and CD8+ T cells and the development of immune memory. During chronic viral infections, there is a balance between virus replication and host immune response, leading to persistence of the virus. On the part of the virus, this often involves suppression of viral lytic genes in favor of viral latency genes. The immune response is often impaired, due to a reduction in host adaptive immune responses and chronic T-cell exhaustion. Chronic viral infections are categorized as either slow, latent, or productive, depending upon the timing of virus replication and the resolution of disease. (Abbreviations: EBV, Epstein–Barr Virus; HBV/HCV/HDV, Hepatitis B/C/D virus; HHV-8, human herpesvirus 8; HIV, human immunodeficiency virus; HPV, Human papillomavirus; HSV-1/2, herpes simplex virus-1/2; HTLV-1, Human T-cell leukemia virus type I; BKV, BK virus; and JCV, John Cunningham virus).

During acute viral infection, a rapid immune response occurs between the infected host and the viral pathogen [1]. Resolution involves either viral clearance and host memory, host death due to overwhelming inflammation and/or extensive viremia, or a transition to a chronic infectious state. Unlike acute viruses, chronic viruses persist in a semi-stable relationship within their host, generating antigenic stimulation for several months to decades. These chronic viral infections can be categorized into: 1- Latent (lack of substantial viral production between initial and late stages); 2- Productive (persistent viral production between beginning and late stages); and 3- Slow infection (increasing viral production from incubation period to late stages) [2] (Figure 1). These stages are established by restricting viral propagation and reprogramming viral gene expression. In conjunction with viral adaptation, the host controls the immune response to prevent overwhelming chronic inflammation that could otherwise become harmful to various tissues.

Enduring hyper-antigenemia (even at low to undetectable levels), which occurs during persistent viral infection, imposes a permanent stress on the immune system [3]. The magnitude of the CD8+ T-cell response following initial infection can be substantial and it is essential that most of the expanded cells die after antigen clearance to maintain lymphoid homeostasis [4]. However, for an efficient memory pool to persist, selected CD8+ T cells that have escaped apoptosis must retain sufficient replicative potential to allow successive rounds of proliferation in response to antigen recall throughout the host’s life. Unlike normal memory T cells, which persist due to the levels of interleukin-7 (IL-7) and IL-15, exhausted T cells only require the presence of viral antigen to continue proliferating [5]. This is partly due to losses in interleukin-2 receptor-β (CD122) and interleukin-7 receptor (CD127) that limit generation of virus-specific T cells [6,7]. Because viral antigen is intermittently or constantly supplied to these cells, viral-specific T cells never cease proliferating. Depending on the length of infection, this could result in progressively shorter telomeres and an age-related decline in T-cell responses.

**Figure 2:**
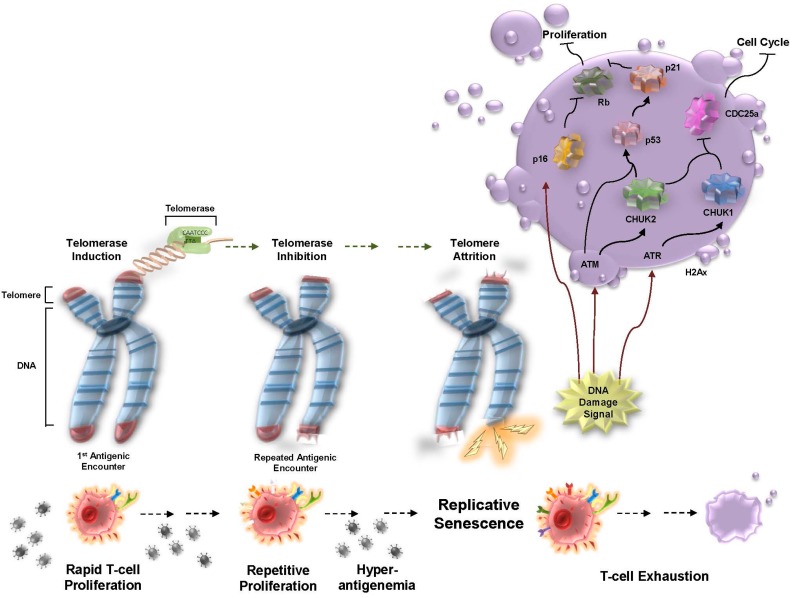
Telomere attrition and the DNA damage response during chronic viral infection. During an initial viral encounter, a robust T-cell response occurs followed by telomerase activation and retention of telomere lengths. Subsequent antigen encounters lead to inactivation of the telomerase promoter and loss of telomerase expression. Telomere attrition, caused by chronic exposure to viral antigen, is exacerbated over time (dotted arrows). Enduring hyper-antigenemia results in telomere crisis and the activation of the DNA damage signal. This results in T-cell exhaustion, inhibition of T-cell proliferation, and cell cycle arrest. The eventual outcome is programmed cell death (apoptosis) or replicative senescence. (Abbreviations: ATM, ATM Serine/Threonine Kinase; ATR, ATR Serine/Threonine Kinase; CDC25a, Cell Division Cycle 25A; CHUK-1/2, Conserved Helix-Loop-Helix Ubiquitous Kinase-1/2; H2AX, H2A Histone Family Member X; p16, Cyclin Dependent Kinase Inhibitor 2A; p21, Cyclin Dependent Kinase Inhibitor 1A; p53, Tumor Protein P53; and Rb, Retinoblastoma 1)

The average telomere length for naive CD4+ and CD8+ T cells is about 2.5 kb longer than effector or memory T cells [8]. It would be easy to infer then that during clonal expansion, memory T cells are at a distinct replicative disadvantage compared to early effector T cells due to a theoretical loss of telomere sequence following the initial encounter with antigen. However, this does not appear to be the case. Antigen-specific T and B cells can up-regulate telomerase activity during the initial response to acute infection, thereby preserving the clonal potential of initial memory T cells for subsequent encounter [9,10]. However, despite the preservation of telomere length, telomerase activity is not retained after resolution of acute viral infection [11]. Enduring antigen-specific T cells are then susceptible to telomere defects, including senescence and death, during subsequent clonal expansions or proliferation (Figure 2). On average, peripheral blood mononuclear cells (PBMCs) lose about 50 bp of telomeric DNA per year, but this loss has the potential to be increased during chronic viral infection, such as in the case of patients receiving antiretroviral treatment (ART) for HIV-1, where loss of up to 250 bp per year of telomeric DNA has been documented [12].

Somatic cells, like those of naïve, effector, and memory T cells, have a finite proliferative capacity, due largely to the inability of DNA polymerase to replicate the distal ends of chromosomes. Pluripotent stem cells and the majority of cancer cells can circumvent this problem by up-regulating telomerase, a reverse transcriptase that can retrogradely add a six base pair (TTAGGG) nucleotide sequence onto the ends of chromosomes [13]. Cellular replication in the absence of telomerase expression results in progressive telomere shortening. Elegant studies demonstrated that a single dysfunctional telomere is sufficient to cause telomere end replication problems and increase chromosomal instability, degradation, and fusion [14,15]. Dysfunctional telomere-induced foci (TIF) engage a DNA damage response (DDR) and trigger activation of the ATM Serine/Threonine Kinase (ATM)/p53-dependent senescence program (Figure 2) [16]. Unlike most somatic cells, T cells, which include CD8+, CD4+, naïve, and memory, can reactivate telomerase through mitogenic stimulation [17,18,19]. These cells initially harbor nearly undetectable levels of telomerase. Upon antigenic stimulation, as is the case with acute viral infection, telomerase is reactivated. This process is reiterated during second antigenic stimulation, but by the third and all subsequent stimulations, T cells are less responsive to mitogenic stimulation and the telomerase gene promoter is inactivated [20].

A deleterious consequence of excessive telomere shortening is the premature induction of replicative senescence of CD8+ T cells [21]. While senescent cells are unable to expand, they can survive for extended periods of time, occupying immunological space where functional immune cells could exist [22]. The accumulation of senescent CD8+ T cells has been proposed to play a role in failed immune surveillance and in facilitating the development of metastasis of certain cancer types [23]. Interestingly, some studies proposed that it may be possible to reverse this phenotype by reactivating telomerase expression [23,24].

Immune senescent T cells bear several trademarks: shortened telomeres, decreased T-cell-specific surface glycoprotein-CD28 (CD28) and T-cell activation antigen CD27 (CD27), co-receptors for T-cell activation, increased β-1, 3-Glucuronyltransferase-1 (CD57), and loss of proliferation [25,26,27]. While CD57+ cells may not be terminally differentiated, it does serve as a marker for highly differentiated CD8+ T cells [28]. CD57+ cells have usually undergone more cell divisions, have an increased risk for activation-induced cell death, and are increased in individuals with chronic immune activation [28]. It is believed that either phenotype, CD57+ or CD28−, can lead to senescence and a loss of replication in some of the cells bearing these markers [27]. The fact that these hallmarks of senescence increase with age and are seen on lymphocytes of healthy, elderly individuals suggests a diminished replication potential of these cells. CD28 loss often precludes CD27 expression, which marks a more terminally differentiated cell. An accumulation of CD8+CD28−CD27− cells occurs during normal aging and chronic-antigen stimulation. These cells have shortened telomeres and lower telomerase activity than less differentiated T cells [29,30,31]. Loss of CD28 can be delayed in HIV-specific T cells transduced with telomerase, suggesting that telomere dynamics provide some intracellular signal for extinction of CD28 expression [32]. A consequence of accumulating CD8+/CD28− T cells is that they can lose their function, have reduced expression of effector molecules (granzyme B and perforin) and reduced cytotoxic T-lymphocyte (CTL) activity [33]. These cells have been found in various chronic viral infections, such as HIV, Epstein–Barr virus (EBV), and Hepatitis C virus (HCV), with each viral-specific CD8+/CD28− T cell responding with varying degrees of the differentiated phenotype [34]. An additional marker of senescent cells is increased expression of Killer Cell Lectin-Like Receptor G1 (KLRG1). Similar to CD57, KLRG1 is expressed on the surface of NK cells and “antigen-experienced” T cells, and leads to defects in signaling and proliferation in highly differentiated T cells [35].

Critical telomere shortening induces the DNA damage response (DDR), and if the DNA damage is not repaired either programmed cell death (apoptosis) or replicative senescence occur. In the absence of apoptosis, cells harboring dysfunctional telomeres are at risk of developing genomic defects, with numerous chromosomal instabilities, such as DNA deletions, amplifications, and aneuploidy [36,37]. In some T cells progressing to senescence, apoptotic markers have been found, such as low expression of the anti-apoptotic gene, B-Cell CLL/Lymphoma 2 (Bcl-2), and up-regulation of cell death markers, such as Tumor Necrosis Factor Receptor Superfamily Member 6 (CD95), which lead to p53-mediated cell death [38]. As an example, HIV-specific CD8+ T cells that had undergone replicative senescence, defined by loss of proliferative capacity and shorter telomeres, were found to be more sensitive to apoptosis [39]. These cells were not CD28− or C–C chemokine receptor type 7 (CCR7)-deficient, but did harbor CD57 expression, and therefore may not be terminally differentiated. CD57 expression is believed to make cells more susceptible to activation-induced cell death by apoptosis [39]. It appears that, in some cases, replicative senescent T cells do undergo apoptosis. This contradiction can be expounded upon as (1) apoptotic resistance is acquired through altered differentiated states during chronic stimulation, (2) many subpopulations of cells exist within the same individual, and (3) senescent cells can, in some settings, be prone to apoptosis progression [27].

Despite these findings, an enhanced resistance to apoptosis in replicative senescent cells is most often seen. Large populations of terminally differentiated, non-functional T cells are found in elderly humans, indicating that these cells undergo senescence and are not eliminated. Replicative senescence is permanent and cannot be overcome by the addition of Phorbol 12-myristate 13-acetate (PMA)/Ionomycin, interleukin-2 (IL-2), or antigen stimulation [40]. In addition, these cells are often more resistant to apoptotic signals, such as dysregulated Fas-mediated signaling [41,42]. In vitro-based studies have shown that repeat antigenic encounters lead to resistance to CD95 and activation-induced cell death [43]. These cells increased PI3K/AKT signaling through Phosphatase and Tensin Homolog (PTEN) loss, leading to impairment in CD95 activation. Cells exposed long-term to antigen also showed increased expression of Bcl-2-Like Protein-1 (Bcl-xl) and reduced expression of pro-apoptotic proteins Bad and Bax. It is likely that less differentiated states (CD27+) are more prone to apoptosis, while late differentiated cells (CD27−) acquire apoptosis resistance, leading to replicative senescence.

## 2. T-cell Exhaustion in Chronic Viral Infection

**Figure 3:**
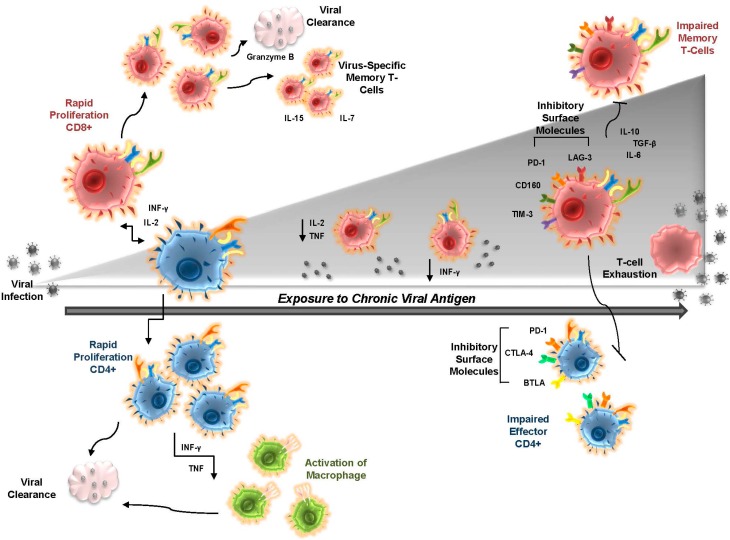
A depiction of T-cell exhaustion during chronic viral antigen exposure. Initial viral encounter leads to rapid activation of host adaptive immunity. A robust CD8+ (red cells) and CD4+ T-cell (blue cells) response occurs, leading to activation (arrows) of immune cytokines (IFN-γ, IL-2, and TNF), proliferation of host immune effector cells (CD4+ and CD8+ T cells, and the activation of macrophages (green cells), natural killer cells, etc), generation of virus-specific memory (responsive to IL-7 and IL-15), and clearance of the invading viral pathogen (increases in perforin and granzymes, for example). Long-term exposure to virus or viral antigen leads to a step-wise decrease in effector cytokines and, over time, the appearance of T cells displaying hallmarks of exhaustion. This includes the up-regulation of inhibitory markers on the surface of the T cell (PD-1, LAG-3, CD160, TIM-3, CTLA-4, and BTLA), a differential loss (IL-2, TNF, and IFN-γ) or gain (IL-10, IL-6, and TGF-β) in cytokine expression, impaired CD8+ and CD4+ T-cell effector response, and impaired memory T cells that are no longer responsive to IL-7 or IL-15. Eventually, this can lead to T-cell senescence and/or apoptosis, rapid viral replication, and disease. (Abbreviations: BTLA, B- and T-Lymphocyte-Associated Protein; CD160, Natural Killer Cell Receptor BY55; CTLA-4, Cytotoxic T-Lymphocyte-Associated Protein 4; INF-γ, interferon-gamma; IL-2, interleukin-2; IL-6, interleukin-6; IL-7, interleukin-7; IL-10, interleukin-10; IL-15, interleukin-15; LAG-3, Lymphocyte Activating 3; PD-1, Programmed Cell Death 1; TGF-β, Transforming Growth Factor β; TIM-3, T-Cell Immunoglobulin Mucin Family Member 3; and TNF, tumor necrosis factor). Arrows show the increase/decrease of cytokine release during antigen exposure and the directionality of the immune response.

T-cell exhaustion is a state whereby normal T-cell functions are impaired as a consequence of chronic antigen stimulation. The details of T-cell exhaustion are still being defined, but include: 1- impaired memory T cells; 2- impaired CD8+ T-cell effector responses; 3- impaired CD4+ T-cell responses; 4- resistance to apoptosis; and 5- accelerated immune senescence (Figure 3). The theory that CD8+ T-cell functions are exhausted during chronic viral infections was originally described during chronic lymphocytic choriomeningitis virus (LCMV) infection in mice [44]. Unlike acute viral infections, some LCMV viral isolates persist within their host and do not cause lethality, thereby mimicking chronic infection [45]. The host’s CTL response disappears following infection with these chronic strains of LCMV. The original proposal advocated that the virus was never effectively cleared because T cells were overwhelmed fighting the virus and the CTL response was eliminated entirely. It is now clear that exhausted T cells show progressive losses in antiviral functions. Proliferative capacity is diminished, followed by sequential loss of interleukin-2 (IL-2) production, cytolytic killing, decreased tumor necrosis factor (TNF) and interferon-gamma (IFNγ) production, and diminished degranulation, all of which can result in clonal deletion [46]. Dampened cytolytic responses during chronic viral infections have been shown in viral-specific CD8+ T cells of HIV, EBV, and cytomegalovirus (CMV) infection [47]. This includes loss of perforin, along with granzyme proteases that are vital for CTL’s ability to eliminate antigen-presenting cells and to control the level of activated CD8+ T cells through apoptosis during chronic infection [48]. There is also an extensive profile of gene dysregulation between exhausted T cells and naïve or effector T cells, including defects in host cell signaling molecules, calcium binding, cytokine signaling, apoptosis/cell death, and migration [6].

Impaired CTL function is attributed, in part, to the presence of co-inhibitory markers that are part of the normal, yet transient, response to acute infection. These inhibitory markers serve to dampen and inhibit further immune responses, but continue to be expressed on the surface of exhausted T cells. A more advanced stage of T-cell exhaustion, marked by damped T-cell effector functions, occurs when more inhibitory markers are found on the surface of the T cell, along with a greater variety of inhibitory markers expressed [49,50]. The most well-known and highly expressed inhibitory marker on exhausted T cells is Programmed Death receptor (PD-1), a member of the CD28 co-stimulatory family. PD-1 modulates the T-cell response to infection, reduces CTL effectors, and decreases proliferation of specific effector memory CD8+ T cells [51,52]. Additional markers found on exhausted T cells include natural-killer cell receptor (2B4), T-cell immunoglobulin and mucin protein-3 (TIM-3), natural killer receptor BY55 (CD160), Ly49 family members, lymphocyte-activation gene 3 (LAG-3), cytotoxic T-lymphocyte-associated protein-4 (CTLA-4), and Prostaglandin E Receptor 4 (Ptger4) [6]. Most of these possess immuno-tyrosine inhibitory motifs or switches that decrease active cell signaling in exhausted T cells. For example, TIM-3 prevents effector TH1 responses and expression of TNF and IFN-γ. This, in turn, has adverse effects on non-T-cell populations, such as dendritic cells, natural killer cells (NKs), and macrophages [53]. It is important to note that studies on inhibitory markers were carried out using the LCMV model in mice. Thirteen inhibitory receptors were found to be differentially regulated on exhausted T cells, which may not represent a complete description in human cells. For instance, the mouse glycoprotein 49B (GP49B) was up-regulated on exhausted T cells, but a human homolog has yet to be identified.

Less is known about T-cell exhaustion in CD4+ T cells. It is clear that exhausted CD4+ T cells have an altered phenotype compared to effector and memory CD4+ T cells. Exhausted CD4+ T cells express similar inhibitory markers and transcriptional factors as their counterpart CD8+ cells, including PD-1, but express a higher ratio of some inhibitory markers such as CTLA-4, CD200, and B- and T-lymphocyte associated protein (BTLA) [54]. Exhausted CD4+ T cells have diminished TH1 responses and altered effector functions, the latter related to their distinct transcriptional program. Regulatory T cells (Tregs) also have altered functions during chronic viral infections. Chronic viral infection leads to up-regulation of PD-1 on the surface of Tregs and enhanced Treg proliferation [55]. This allows for greater inhibitory function against CD4+ and CD8+ T cells. In addition, expression of CTLA-4 on the surface of Tregs aids in enhancing their immune suppressive actions [56,57].

## 3. Telomere Dynamics and T-cell Exhaustion during Chronic/Productive Viral Infections

Exhausted T cells have been found in individuals infected with a multitude of human chronic viruses and in mice with LCMV (as described above). Telomere shortening in viral-specific T cells has a hierarchical ranking system, with some antigens producing telomere shortening at a faster rate than other antigens. CMV infection seems to take the highest spot in this hierarchy, having a more differentiated state and shorter telomeres than any other virus, such as HIV, EBV, and HCV [38]. It has yet to be confirmed, but one could infer that viral infections that lead to a more extensive exhaustive state would be prone to faster telomere shortening and immune senescence.

HIV infects CD4+ T cells and is responsible for acquired immune deficiency syndrome (AIDS). Due to advances in antiretroviral therapy (ART), HIV-infected individuals are living longer. During chronic HIV infection, the immune system bears a striking resemblance to those of otherwise healthy, elderly people, with higher incidences of infection and loss of effector functions [58]. This is despite the fact that there is a precipitous drop in infectious HIV between acute and chronic phases, with over 99% of HIV antigen coming from HIV particles in later stages. This suggests that a consequence of ART is that HIV-specific T cells are exposed to viral antigen for a longer period of time, leading to increases in HIV-specific T-cell exhaustion and possible immune senescence. Indeed, markers of both T-cell exhaustion and immune senescence have been documented in HIV-infected individuals. These include enhanced expression of inhibitory markers (such as PD-1, CTLA-4, and LAG-3), reduction in effector cytokines, reduced proliferative and self-renewal potential, and a lack of CD4+ T-cell help [59,60,61]. In fact, the expression of PD-1 correlates with impaired T-cell function, reduced CD4+ T cells, increased viral load and a sensitizing of the cells to apoptosis [62,63]. This is significant, as HIV-induced apoptosis of uninfected “bystander” cells contributes to HIV-associated T-cell death and disease progression, a process that is not completely understood [64,65]. The significance of the interplay between T-cell exhaustion and HIV disease is demonstrated by the fact that aggressive forms of the disease harbor more cells with advanced T-cell exhaustion and PD-1 therapy reduces the exhausted phenotype, leading to increased T-cell effector functions and a reduction in virus load [66].

While ART treatment can effectively control CD4+ T-cell counts and viremia, an unintended consequence is a rise in CD8+ T-cell levels [67]. These CD8+ T cells have been linked to an increase in complications and non-AIDS-related malignancies. Researchers have discovered that while there is an increase in the immune senescence phenotype (CD8+ T cells that are CD28−/CD27), most of these are CMV-specific. This is due to the fact that, during chronic HIV infection, there is an increase in viral-specific, non-HIV CD8+ T cells. In fact, most HIV-specific CD8+ T cells have an intermediate phenotype in which they still express CD27. Another report determined that an increase in CD57 on HIV-specific CD8+ T cells serves as a better marker of immune senescence since it correlates with replicative capacity [68]. HIV-specific CD8+/CD57+ cells were more susceptible to apoptosis and point to a general trend that in all cells (regardless of infectious state) CD57 expression is indicative of more cellular divisions that result in shorter telomeres.

A major consequence of harboring HIV-exhausted T cells is premature aging. In accordance, immune senescent cells are found in HIV-infected individuals. Telomere lengths in more differentiated cells (CD8+/CD28−) are significantly shorter in HIV-infected than non-infected individuals. These cells have poor replicative potential and are characterized as immunosenescent; however, a determination of HIV specificity was not made. This is a necessity since long-term expanded CD8+/CD28− cells are known to have shorter telomeres and undergo replicative senescence in normal individuals [69]. This phenomenon has also been seen in mature individuals and in children (aged 0–5 years) infected with HIV [70]. These children harbored shorter telomere lengths and had a higher proportion of CD8+ senescent cells. This appeared to be specific to the CD8+ cells, since CD4+ cells were not senescent even though they expressed PD-1 at a higher level. Likewise, a comparison of telomerase and telomere lengths in CD4+ and CD8+ populations from HIV− and HIV+ sets of twins demonstrated increased telomere lengths in CD4+ T cells and decreased telomere lengths in CD8+ T cells [71]. CD4+ T cells in HIV+ twins retained their replicative potential despite the fact that telomerase activity remained low to undetectable in all populations of cells. This suggests that CD4+ T cells do not undergo replicative senescence in HIV infection. However, the fact that CD8+ T cells demonstrated shorter telomeres was in line with T-cell senescence, though in this study (as in the one above), CD8+ T cells were not sorted for HIV-specific memory cells.

Apposite support for telomere shortening in HIV-specific exhausted T cells results from a study characterizing telomere dynamics between HIV progressors and HIV controllers. A reduction in telomere lengths in HIV-specific CD8+ T cells was demonstrated in HIV progressors [72]. In HIV progressors, these HIV-specific CD8+ T cells had reduced telomerase activity and telomere lengths that were shorter not only in comparison to CD8+ T cells specific to HIV controllers, but also to CMV-, EBV-, or bulk CD8+ T cells from the same person. When the PD-1/PD-L1 pathway was blocked, telomerase activity and telomere lengths increased in the HIV-specific CD8+ T cells from progressors. To determine the role of telomere shortening in limiting CD8+ T-cell replication, HIV CD8+ T cells have been transduced with telomerase [32]. Telomerase addition results in increased HIV targeting and a reduction in p16 and p21 levels.

Like HIV, hepatitis B and C (HBV and HCV) viruses are productive, chronic viruses and display T-cell exhaustion from telomere shortening. HBV and HCV are the major causes of acute liver infection; however, only HBV and HCV are responsible for a large proportion of global, chronic liver disease. Anywhere from 60–90% of HBV and HCV infections reroute to chronic infection. A delayed immune response to the ever-changing viral mutational landscape allows the virus to adapt and persist, especially in HCV infections. In fact, chronic viral liver disease is nearly completely due to the adaptive immune response where immunopathology, consisting of a low-level CTL response, destroys hepatocytes and leads to liver cirrhosis and hepatocellular carcinoma (HCC) [69].

Telomere length measurements in HBV and HCV infection have been performed in the host cell hepatocytes. Telomere lengths have been found to be shorter in hepatocytes from chronic HCV infection, especially in HCC, which leads to chromosomal instability [73,74,75]. Studies on replicative senescence in HCV-specific CD8+ T cells are difficult due to the relative scarcity of HCV-specific cells in the periphery [76]. Despite this difficulty, an abundance of data supports the notion that telomere attrition occurs in total T cells from chronic HBV and HCV infections. Telomerase RNA levels were found to be lower in the peripheral blood lymphocytes (PBLs) of HBV- and HCV-infected individuals that may contribute to immunosuppression [77]. Telomere length assessments in HCC patients infected with HBV or HCV demonstrated shorter telomere lengths not only in liver biopsied tissue, but also in PBLs [78]. Telomere attrition was not seen in HBV/HCV-noninfectious HCC. This suggests that long-term HBV/HCV infection causes adverse effects on telomere lengths in PBLs. In support of this, chronic HCV patients have shorter telomeres in CD4+ and CD8+ T cells with the memory phenotype [79]. Telomere lengths were comparable to those of normal aging of 10 years, which was enhanced to 15 years with more severe fibrosis disease [79]. Telomere shortening has not been found in cell-free serum DNA from chronic HBV infections [80].

When telomere lengths were followed over 15 years in individuals infected with HCV or dual HCV/HIV, researchers found differences in telomere lengths compared to lengths of non-HCV-infected individuals [81]. Telomere lengths in CD4+ T cells were shorter in HCV-infected individuals and shorter in both CD4+ and CD8+ T cells in dual infection. Telomere lengths did not correlate with a more differentiated cell, again owing to the possibility that length measurements were taken on total PBMCs from the patients and were not sorted for viral-specific T cells. Among the genes altered during chronic HBV infection was telomeric repeat binding factor 2, interacting protein (TERF2IP) [82]. TERF2IP is known to regulate telomere dynamics and TERF2IP expression was decreased in HBV-specific CD8+ T cells. In addition, expression of genes associated with cellular senescence were found down-regulated in exhausted HBV-specific CD8+ T cells, including ATM, several DNA repair proteins (RAD proteins), and POLH (DNA Polymerase Eta), a polymerase involved in translesion repair [79].

Early exposure in life to HBV infection can possibly lead to shorter telomere lengths in CD8+ T cells and early development of immunosenescence [83]. While only an indirect measurement of HBV infection was performed, these individuals lived in HBV endemic areas where HCC development was high and had T-cell immune markers of HBV infection (TCRVβ12). These individuals also demonstrated low CD3+ and CD4+ T-cell counts, suggesting immune impairment from long-term exposure to HBV. Chronic HCV patient CD8+ T cells were shown to have shorter telomeres with hallmarks of DNA damage, including γ-H2AX (marker of DNA double-stranded breaks) and p53 serine phosphorylation [84]. Immune impairment was seen in the CD8+y-H2AX+ T cells, with failed signaling through the JAK/STAT signaling pathway. The majority of T cells that were y-H2AX-positive were highly differentiated, with either CD27−CD57− or CD27−CD57+ phenotype, but did not express PD-1 or TIM-3, possibly owing to the fact that HCV-specific CD8+ T cells were not analyzed. It could be that these T cells, since not HCV-specific, could owe their phenotypes to bystander effects from secreted pro-differentiation factors released during the course of the chronic viral infection. This has been described for CMV infection, in which release of interferon-alpha (IFN-α) causes CMV-specific CD4+ T cells to differentiate and lose telomere lengths [85]. IFN-α can be released in response to CMV infection and inhibits telomerase activity, leading to telomere shortening [86]. So, while a direct examination of telomere lengths and senescence has not been examined for HBV- (or HCV-) specific CD8+ T cells, it can be suggested that telomere dynamics and slowed replication are altered in exhausted HBV-specific T cells.

Exhausted viral-specific T cells have been described for both HBV and HCV. Exhausted HCV-specific T cells have been found in the liver, spleen, and blood, and the exhausted inhibitory receptor phenotype varies according to the inhibitory marker and level of expression. CD127, or the IL-7 receptor, is up-regulated on effector and memory T cells, and is a marker for the activated state during HBV acute infection [87]. Low CD127 expression is seen on exhausted CD8+ T cells in other chronic viral infections, such as HIV [88]. In HCV-specific T cells that expressed low CD127 and high inhibitory markers, impaired proliferation was seen and could only be reinvigorated once PD-1/PD-L1 was blocked. In addition, blockade of TIM-3 enhanced T-cell proliferation and CTL responses [89]. Not only was TIM-3 present on the surface of chronically infected HCV T cells, but CD57 was also present in conjunction with TIM-3 [89]. Given the co-localization of these two markers on the same cell, it suggests that during chronic HCV infection, exhausted T cells undergo senescence.

## 4. Telomere Dynamics and T-cell Exhaustion during Chronic/Latent Viral Infections

Less is axiomatic between viral-specific T-cell exhaustion in latent viral infections, including whether or not latent viral infection can lead to shortened telomeres, replicative senescence, and/or apoptosis. The majority of studies on T-cell exhaustion were performed in the LCMV model, which, though correctly depicting chronic productive viruses, does not accurately reflect chronic latent viruses. Often, these viruses are hidden from immune surveillance and/or produce a low abundance of viral antigens. Studies using an adapted influenza A virus to mimic chronic antigen stimulation have shown that CD8+ T-cell exhaustion still occurs [90]. This model allowed for the absence of large viral loads and lymphocyte infection, and demonstrated T-cell exhaustion, albeit with altered mechanisms. While evidence of T-cell exhaustion and/or senescence is found in latent chronic viral infections (as outlined below), there is still a large gap in research describing telomere dynamics during these infections. A majority of the chronic latent viruses discussed are known to be oncogenic, and have demonstrated altered telomerase activity and shorter telomeres in the infected cell [91]. However, only EBV studies have demonstrated telomere attrition in EBV-specific T cells, highlighting the fact that more studies need to be done to examine the relationship between long-term, chronic viral infections and their effect on viral-specific immune responses in regard to telomere attrition and senescence.

An additional hindrance to the study of T-cell exhaustion and its downstream effects during latent viral infection is the lack of data owing to the endemic nature of some of these viruses. A challenge remains in finding non-infected T cells for some worldwide endemic latent viruses. For example, in order to measure T-cell exhaustion or telomere lengths in varicella zoster virus (VZV)-specific T cells, non-VZV T cells are required for comparison. The estimate for the number of infected individuals with VZV is over 90% in temperate climates without vaccination. Therefore, finding individuals without long-term VZV circulating T cells is difficult. Despite these constraints, hallmarks of T-cell exhaustion have been found in two characteristic latent viruses, VZV and herpes-simplex-1/2 virus (HSV-1/2). VZV-specific T cells declined with age, lost T-cell functions, and expressed T-cell exhaustion markers [92,93]. In vivo models have also demonstrated the existence of HSV T-cell exhaustion in CD8+ T cells [94]. When compared over time, telomere lengths in VZV- and HSV-specific T cells were not significantly shortened or senescent. This observation may result from the fact that VZV does not present as a major antigenic attractant due to its high ability to be bypassed by the immune system and the location of latent viral reservoirs [95]. In one patient, VZV-specific T-cell proliferation and telomere lengths actually increased during a period of shingles [95]. Though this was a single case, a larger study may demonstrate that reactivation of the virus from latency can induce telomere elongation in these cells or, more likely, that newly activated naïve effector T cells (with longer telomeres) are converted to new memory cells.

While VZV and HSV are effectively hidden from immune surveillance due to their location in neurons, human T-cell leukemia virus type-1 (HTLV-I) infects cells of the immune system, therefore the ability to avoid immune surveillance is limited. In addition, unlike VZV, HTLV-I is not endemic in the population. HTLV-I, like HIV, is a retrovirus that infects predominantly CD4+ T cells and is responsible for two very different diseases: an invariably fatal T-cell cancer, Adult T-cell Leukemia/lymphoma (ATLL), and a neurodegenerative disease, tropical spastic paraparesis/HTLV-I-associated myelopathy (TSP/HAM). ATLL develops after a very long latency period of several decades in individuals infected with HTLV-I, while the latency period is shorter for TSP/HAM [96]. While a direct examination of telomere lengths in HTLV-I-specific CD8+ T cells has not been performed, several pieces of data (described below) argue that replicative senescence is occurring in these cells. This includes the long latency period of HTLV-I, the up-regulation of inhibitory markers, the loss of effector T-cell function, and a more differentiated/senescent phenotype seen in HTLV-I cells.

Profound T-cell dysfunction has been found in HTLV-specific CD8+ T cells. HTLV-I-specific CD8+ T cells have been found in HTLV carriers and ATL and TSP patients [97,98,99]. The prevailing theory is that a dysfunctional CTL response against the HTLV virus, in particular the immunodominant viral antigen Tax, may lead to reduced elimination of HTLV-I cells from the host and ATL progression [100,101]. In the case of TSP, reduced CTL function may promote disease symptoms. HTLV-I-infected patients demonstrate a lack of recall response to highly immunogenic antigens in TSP/HAM but not in HTLV-I-infected asymptomatic carriers [100]. HTLV-I-(Tax)-specific CD8+ T cells were found in carriers, TSP/HAM, and ATL patients; however, ATL patients demonstrated significantly less Tax-specific CD8+ T cells than other viral states [101]. When stimulated ex vivo with Tax, TSP/HAM patients and carriers were able to proliferate and produce IFN-γ; however, proliferation was severely impaired in ATL and a small percentage of HTLV-I carriers. In fact, up to 10% of viral-specific T cells were found to have lower CTL activity, such as lower perforin and granzyme B, along with lower expression of immune co-stimulatory molecules in TSP patients [102]. In the case of ATL, profound immune disruption was found in ATL patients, including reduced CD8+ T cells harboring epitopes to more immunogenic viral antigens and lower CTL activity [100,103].

An increase in T-cell exhaustion markers has been reported in HTLV-I-infected cells from HTLV-I carriers and diseased individuals [99,104]. HTLV-I viral proteins can also increase the expression of inhibitory molecules. The expression of the co-inhibitory marker, T cell immunoglobulin and ITIM domain (TIGIT), was increased by viral proteins and suppressed T-cell responses [105]. TIGIT was found to be expressed on cells from ATL or TSP patients, and blocking either TIGIT or PD-1 led to enhanced immune response to HTLV-I in TSP patients. Along with PD-1 and TIGIT, other inhibitory markers have been demonstrated to play a function in HTLV-I-infected cells, including TIM-2 and 2B4 [100,104,106,107]. Blocking the PD-1/PD-L1 pathway in cells led to increases in T-cell cytotoxic functions, including enhanced INF-γ and TNF-α expression. Inhibition of 2B4 signaling resulted in improvement in the function of HTLV-I-specific CD8 T cells, with increased perforin expression. TSP/HAM patients also displayed higher levels of 2B4 on CD8+ T cells, but they had an opposite function and inhibited de-granulation [108].

T cells in TSP patients were also deficient in CD28 and CD27 expression, the latter indicative of terminal differentiation. Over 60% of HTLV-specific CD8+ T cells in TSP patients expressed the late differentiated phenotype (CD28−/CD27−), whereas 60% of HTLV-specific CD8+ T cells in HTLV carriers had the intermediate phenotype (CD28−/CD27+) [102]. Several studies have reported characteristics associated with a senescent phenotype during HTLV-I infection: impaired CD8+ T cells in their ability to proliferate in response to antigen stimulation, increased levels of pro-inflammatory and pro-senescence cytokines such as IL-6 and TNFα, and reduced expression of CD28 [109]. In vitro, Tax-specific T cells from TSP/HAM patients also display phenotypes of effector memory T cells (CD45RA+/CCR7−) and terminally differentiated effector memory cells (CD45RA−/CCR7) [110].

ATL patients are known to harbor shortened telomeres and have defects in telomerase expression in total PBMCs [111]. The short telomeres in HTLV-I-infected cells are recognized as DNA double-strand breaks and activate the DDR pathway, elevating ATM and p53 activities, culminating in replicative senescence [112]. However, the role of telomere dysfunction and senescence in HTLV-specific CD8+ T cells has never been investigated in these patients. Whether the senescent phenotypes seen in HTLV-I diseases were due to telomere attrition or something else (such as oxidative damage) has not been studied in HTLV-I-specific viral memory cells.

Similar to HTLV-I, Epstein–Barr virus (EBV) is a chronic, latent virus that is oncogenic and linked to several cancers. It is estimated that nearly 90% of the world population is infected with EBV. EBV is responsible for a wide plethora of human diseases, including those associated with defective B cells, such as acute infectious mononucleosis (AIM), and a wide variety of non-B-cell diseases. Given the prevalence of EBV infection in the population, it is likely that most individuals harbor EBV-specific CD8+ T cells. Studies have shown PD-1 expression on EBV CD8+ T cells in healthy individuals, which were increased in EBV CD8+ T cells from EBV mono-infection or EBV/HIV co-infection. High expression of TIM-3 was also found on EBV CD8+ T cells in EBV/HIV co-infected individuals [113]. The level of PD-1 expression on EBV CD8+ T cells was nearly equal to that of HIV CD8+ T cells. In EBV+ diffuse large B-cell lymphoma (DLBCL), administration of PD-L1 caused enhanced proliferation and cytokine expression, and restored antitumor immunity [114]. EBV exhausted T cells have also been linked to the emergence of several conditions and a wide variety of EBV-associated diseases. This includes chronic fatigue syndrome (CFS), multiple sclerosis (MS), and systemic lupus erythematosus (SLE). Signs of EBV-specific T-cell exhaustion included elevated PD-1 and decreased cytotoxic T-cell functions in EBV CD8+ T cells in SLE, diminished T- and B-cell memory response in CFS, and reduced EBV CD8+ T-cell functionality and exhaustion in MS [115,116,117]. PD-L1 has also been found to be elevated in EBV-positive HL and PTLD [118]. In the latter, it was shown that an EBV latent gene product, LMP1, could elevate PD-L1 gene promoter expression.

A clearer understanding of the effect of telomere dynamics during chronic EBV infection came from studying EBV-specific CD8+ T cells during and after AIM. During and immediately following AIM, EBV-specific CD8+ T cells were able to prevent telomere erosion by up-regulating telomerase [19]. In the majority of individuals, telomere lengths remained unchanged immediately following AIM; however, in longitudinal studies in infected patients (15 months to 14 years), a marked telomere attrition was noted in EBV-specific CD8+ T cells [19]. These cells continued to be exposed to EBV antigen but were unable to up-regulate telomerase. This, and the fact that long-term clones of EBV-specific T cells decrease over time, suggest that chronic EBV exposure leads to replicative senescence of EBV CD8+ T cells [119].

In other situations in which EBV infection is not controlled, researchers examined telomere dynamics. Chronic EBV infection and replication often occurs in HIV-infected immunocompromised individuals. In fact, AIDS-related non-Hodgkin lymphoma (AIDS-NHL) is thought to evolve due to accumulation of EBV-specific T cells that have lost their cytotoxic immune function (i.e., exhausted) and are no longer able to control EBV infection [120]. In these individuals, EBV-specific CD8+ T cells showed marked decreases in telomere length, which seemed to preclude a senescent-like phenotype towards EBV infection, as suggested due to a large decrease in IFN-γ-producing EBV CD8+ T cells, which was similarly seen in the elderly [121]. A similar situation occurs in X-linked lymphoproliferative syndrome (XLP), in which there is an inflated immune response to EBV infection. An examination of EBV-specific CD8+ T cells in survivors demonstrated loss of telomerase, shorter telomere lengths, and a more differentiated state (CCR7−/CD27−) [122].

Unlike HTLV and EBV infections, there is little to no information regarding telomere shortening and how it relates to T-cell exhaustion during some chronic viral infections. This is the case for chronic Kaposi’s sarcoma-associated herpesvirus (KSHV) infection (also known as human herpesvirus-8 (HHV-8)) and human papilloma virus (HPV) infection. To date, no study has been done to examine T-cell exhaustion or senescent markers, nor telomere length on HHV-8-specific memory cells. Given the fact that T-cell exhaustion has been found in all chronic viral infections, it is conceivable that this is also the case in HHV-8 patients. Patients with Kaposi’s sarcoma (KS) had lower amounts of HHV-8-specific CD8+ T cells than HHV-8 carriers [123]. This was seen in both HIV-related KS and classic KS, suggesting global HHV-8-specific T-cell dysfunction in diseased patients. In addition, PD-1 was expressed on natural killer cells (NK) of KS patients and PD-L1 staining was also found on some cells of HHV-8-associated diseases [124,125]. Senescent markers, such as decreased CD57+/CD28− on CD4+ and CD8+ T cells, have also been found in HHV-8-infected individuals with HIV-associated KS [126].

Information is also needed on replicative senescence and T-cell exhaustion in chronic HPV infection. However, the low antigenic loads of HPV infection likely contribute to the fact that T-cell exhaustion may not occur to the same extent, or is difficult to detect, in HPV-infected individuals compared to other chronic viral diseases. This is because long-term existing T cells require the presence of viral antigen to survive and the direct amount of antigen dictates T-cell exhaustion [127]. HPV favors the use of non-preferred codons to reduce the frequency of HPV virus and antigen that is exposed to the immune system [128]. Indeed, HPV-specific T cells are absent or in negligible numbers in the peripheral blood of patients with cervical cancer [129]. This may result from the absence of T cells in the infected area or cross-reactivity of HPV epitopes with other pathogens [130]. An additional consideration is that for some chronic latent viruses, viral-specific CD8+ T cells may be located outside the blood stream. In the case of HPV, HPV-specific CD8+ T cells may be absent from peripheral blood and instead be localized in mucosal tissues [131,132]. Therefore, telomere length measurements and T-cell exhaustion markers may need to be analyzed on memory CD8+ T cells from the mucosa. There are some reported studies suggesting T-cell dysfunction; however, most studies have not directly assessed the phenotype of HPV-specific CD8+ T cells, i.e., inhibitory markers or cytokine profile. T cells and dendritic cells in cervical tissue were positive for PD-1 in high-grade HPV infection [133]. This was seen in conjunction with a reduction in IFN-γ and IL-12 from helper T cells. It has also been shown in HPV-associated recurrent respiratory papillomatosis (RRP) that HPV-specific T cells are dysfunctional, with decreased STAT5 activity and reduced IL-2; however, this has been linked to T-cell anergy and not exhaustion, as these cells did not express PD-1 [134].

PD-1 therapy was able to boost vaccines directed against HPV16’s E6 and E7 genes by boosting tumor-infiltrating CD8+ T cells [135]. HPV-associated head and neck squamous cell carcinoma (HNSCC) is generally more responsive to chemotherapy than HPV-negative HNSCCs. It was found that HPV+ HNSCCs had increased PD-1 expression on infiltrating T cells and that in mice given vaccine therapy, PD-1 expression was elevated and had a more favorable outcome [136]. However, these cells were likely not exhausted, since over half of them did not express TIM-3.

The HPV virus has been shown to alter telomere dynamics. The E6 and E7 viral proteins of HPV can induce or augment telomerase expression, respectively [137,138]. This can lead to telomere attrition and enhanced chromosomal instability in infected cells [139]. This was seen in cervical intraepithelial neoplasia (CIN) patients wherein telomere lengths were significantly shorter in all stages of disease and activated the DDR pathway [140]. Though HPV-specific CD8+ telomere dynamics have not been studied, several reports demonstrate telomere attrition in the PBLs of HPV-infected individuals. HPV-infected individuals with shorter telomere lengths in their PBLs were at a greater risk of developing oropharyngeal squamous cell carcinoma (OSC) and having OPC tumors, and HPV seropositivity led to a correlation with shorter telomere lengths in the PBLs of esophageal squamous cell carcinoma (ESCC) [141,142]. PBLs from head and neck cancer patients were also shown to harbor shortened telomeres; however, a distinction between HPV+ or HPV− was never made [143].

## 5. Concluding Remarks

Evidence is mounting that high levels of antigen stimulation result in excessive proliferation, driving cells into a state of replicative senescence due to telomere attrition. The benefits for addressing viral T-cell exhaustion and immune senescence in patients with chronic viral infections and chronic inflammatory or auto-immune diseases are great so as to finally eradicate the chronic virus. Therefore, it is relevant to the ongoing efforts to develop therapeutic vaccines aimed at stimulating CD8+ T-cell responses and current immunotherapy based on adoptive transfer of expanded virus-specific CD8+ T cells.

There are still many questions when it comes to the therapeutic potential of blocking T-cell exhaustion. One concern is whether fully exhausted T cells can be reactivated. If exhausted T cells have reached a state of terminal differentiation, they may have undergone permanent cell cycle arrest and irreversible cellular senescence. In this case, it is important that anti-exhaustion therapy (such as drugs to block immune inhibitory markers, as discussed below) be given at the proper time, before the cells become permanently differentiated. In the latter case, it would then be imperative to target these cells for removal through enhanced cell death, since reactivation is not possible. Furthermore, in a subset of CD8+ T cells exposed to chronic viral infection, expression of the T-cell factor-1 (TCF-1) gene has been shown to be elevated [144]. Because these cells retain properties of both exhausted and memory T cells, TCF-1+ cells may represent a pole of cells that can be reactivated and proliferate during chronic infection, when inhibitory markers (such as PD-1) are blocked.

Another concern is whether anti-exhaustion therapy can fully reactivate viral-specific pools of memory T cells and whether exhausted T cells retain all their effector functions. Another caveat is determining how many and which immune inhibitory receptors should be targeted. As shown above, the extent of up-regulated inhibitory markers on T cells varies between viruses, so it would be easy to infer that different therapies would be more or less effective for different viruses. For example, a drug targeting CTL-4, ipilimumab, was approved by the Food and Drug Administration (FDA) and has shown success in patients with metastatic melanoma. However, in some viral infections, targeting CTLA-4 has led to increased viral production and a reduction in anti-viral therapy [145]. In chronic HCV infection, PD-1 therapy led to only small increases in proliferation, suggesting that a cocktail of therapies against various inhibitory markers would be more successful. Use of an anti-PD-1 antibody had great effects in the simian version of HIV (simian immunodeficiency virus—SIV). SIV CD8+ T cells increased in the blood and led to enhanced proliferation and effector functions and a drop in SIV viral levels [146]. PD-1 was also shown to be effective in an in vivo model of HBV, whereby blocking PD-1/PD-L1 interactions led to increased IFN-γ and enhanced effector functions that cleared the HBV virus [147]. The FDA has already approved two anti-PD-1 therapies, pembrolizumab and nivolumab, for use in metastatic melanoma [148]. Their use has not yet been approved for chronic viral infections but there are ongoing clinical trials, such as the use of nivolumab for treating HTLV-I-associated ATL. In addition, a phase 2 trial for nivolumab on HPV-associated squamous cell carcinoma of the anal canal (SCCA) patients showed complete or partial responses in some patients [149]. A combination of anti-PD-1 and anti-CTLA-4 therapies produced an enhanced ability to control EBV infection, leading to reductions in lymphomas, increased EBV effector responses, and decreases in EBV-infected B cells [150]. Additional evidence for blocking more than one inhibitory receptor is seen in cancers, such as non-small cell lung cancer patients that develop resistance to anti-PD-1 therapy [151]. This has been linked to an enhancement of TIM-3 on the cell surface of PD-1-resistant cells.

One target that could prove promising is P-Selectin Glycoprotein Ligand 1, PSGL-1. Knockdown of PSGL-1 led to improved anti-viral response and down-regulation of not only PD-1, but several inhibitory receptors, including CD160, BTLA, TIM-3, and LAG-3 [152]. Blockade of PSGL-1 could therefore be a more effective therapeutic target against T-cell exhaustion than targeting individual inhibitory markers alone. Since telomere attrition appears linked with long-term T-cell exhaustion, another concern to address is whether telomerase therapy needs to be added in addition to anti-exhaustion therapy. Rebounding exhausted T cells would still have critically shortened telomeres that are unable to up-regulate telomerase. Telomerase may be required to restore the proliferative capacity of the newly effective T cells.

Furthermore, there are a few concerns that will need to be addressed in upcoming studies regarding T-cell exhaustion and immune senescence: 1- An important limitation of studies previously performed in some chronic viral infections is the use of total PBMCs instead of specific sub-populations and the use of Southern blotting of telomere restriction fragments (TRF) to measure telomere lengths. These assays give a general overview but do not allow distinction between different cellular subtypes and individual chromosomes. This is important because a single chromosome harboring a critically short telomere can initiate senescence [9]. Studies will need to be repeated to look at the individual chromosome level. 2- Most of the molecular determinants for T-cell exhaustion have been discovered with the use of LCMV infection in mice or have been interpreted from viral studies carried out in mice. However, mice have extremely different telomere dynamics than humans. For one, unlike humans, mice have extremely long telomeres [153]. A second concern is the lifespan differences between the two species. Humans live considerably longer (over 80 years) than mice (2–3 years), and therefore the effects of progressive T-cell exhaustion, telomere shortening, and senescence should be far more pronounced and dramatic in humans [154]. 3- Given the shorter lifespan and often sterile experimental laboratory conditions, mice are not exposed to as many distinct pathogens as humans are. Therefore, mice do not have as many distinct, viral-specific, long-term memory T cells as humans. 4- HIV and HTLV are retroviruses that require reverse transcriptase for viral production [155]. Most retroviral therapies, consequently, would also target telomerase. In these cases, the telomere shortening effect could be even greater than in other viral infections that do not rely upon a reverse transcriptase for proliferation.

An additional consideration to keep in mind is that while telomerase loss and telomere shortening have been described in immune senescence, the molecular mechanisms responsible for shutting down telomerase expression have not been elucidated. It is intriguing that repeated cellular stimulation in immune cells results in transcriptional silencing of the telomerase promoter. Therefore, while the outcome (telomere shortening and immune senescence) may be similar in chronic infections, the cell sub-populations and the underlying signaling mechanisms involved are worth investigating. Understanding the regulatory mechanisms controlling telomerase expression in chronic infection will allow the design of specific targeted therapies and may apply to some other chronic stimulation disorders.

In conclusion, sustained antigenic stimulation due to chronic viral infection imposes considerable constraints on the immune system. Chronic proliferation of viral-specific memory T cells can lead to telomere shortening, activation of the DDR, and replicative senescence. T-cell exhaustion occurs, leading to loss of CTL effector and memory cell function. These constraints make it difficult to effectively clear the virus and can lead to re-activation of the virus later in life, cancer (as is the case for oncogenic viruses), disease reemergence, and host death, making alleviation of T-cell exhaustion a priority in antiviral therapy.
